# A phase II randomized controlled trial of three exercise delivery methods in men with prostate cancer on androgen deprivation therapy

**DOI:** 10.1186/s12885-018-5189-5

**Published:** 2019-01-03

**Authors:** Shabbir M. H. Alibhai, Daniel Santa Mina, Paul Ritvo, George Tomlinson, Catherine Sabiston, Murray Krahn, Sara Durbano, Andrew Matthew, Padraig Warde, Meagan O’Neill, Narhari Timilshina, Roanne Segal, Nicole Culos-Reed

**Affiliations:** 1 University Health Network, University of Toronto, Toronto, ON M5G 2C4 Canada; 2 Cancer Care Ontario, York University, Toronto, ON M3J 1P3 Canada; 3 University of Toronto, Toronto, ON M5S 2J7 Canada; 4 The Ottawa Hospital Cancer Centre, University of Ottawa, Ottawa, ON K1N 6N5 Canada; 5 University of Calgary, Calgary, AB T2N 1N4 Canada; 6 Toronto General Hospital, 200 Elizabeth St Room EN14-214, Toronto, Ontario M5G 2C4 Canada

**Keywords:** Androgen deprivation therapy, Cost-effectiveness, Exercise, Fatigue, Patient adherence, Physical fitness, Prostate cancer, Quality of life, Randomized controlled trial

## Abstract

**Background:**

Existing evidence demonstrates that 1:1 personal training (PT) improves many adverse effects of androgen deprivation therapy (ADT). Whether less resource-intensive exercise delivery models are as effective remains to be established. We determined the feasibility of conducting a multi-center non-inferiority randomized controlled trial comparing PT with supervised group (GROUP) and home-based (HOME) exercise programs, and obtained preliminary efficacy estimates for GROUP and HOME compared to PT on quality of life (QOL) and physical fitness.

**Methods:**

Men with prostate cancer on ADT were recruited from one of two experienced Canadian centres and randomized 1:1:1 to PT, GROUP, or HOME. Randomization was stratified by length of ADT use and site. Participants completed moderate intensity aerobic and resistance exercises 4–5 days per week for 6 months with a target 150 min per week of exercise. Exercise prescriptions were individualized and progressed throughout the trial. Feasibility endpoints included recruitment, retention, adherence, and participant satisfaction. The efficacy endpoints QOL, fatigue, and fitness (VO2 peak, grip strength, and timed chair stands) in GROUP and HOME were compared for non-inferiority to PT. Descriptive analyses were used for feasibility endpoints. Between-group differences for efficacy endpoints were examined using Bayesian linear mixed effects models.

**Results:**

Fifty-nine participants (mean age 69.9 years) were enrolled. The recruitment rate was 25.4% and recruitment was slower than projected. Retention was 71.2%. Exercise adherence as measured through attendance was high for supervised sessions but under 50% by self-report and accelerometry. Satisfaction was high and there was no difference in this measure between all three groups. Between-group differences (comparing both GROUP and HOME to PT) were smaller than the minimum clinically important difference on most measures of QOL, fatigue, and fitness. However, two of six outcomes for GROUP and four of six outcomes for HOME had a > 20% probability of being inferior for GROUP.

**Conclusions:**

Feasibility endpoints were generally met. Both GROUP and HOME interventions in men with PC on ADT appeared to be similar to PT for multiple efficacy outcomes, although conclusions are limited by a small sample size and cost considerations have not been incorporated. Efforts need to be targeted to improving recruitment and adherence. A larger trial is warranted.

**Trial registration:**

**ClinicalTrials.gov:** NCT02046837. Date of registration: January 20, 2014.

**Electronic supplementary material:**

The online version of this article (10.1186/s12885-018-5189-5) contains supplementary material, which is available to authorized users.

## Background

Nearly half of men diagnosed with prostate cancer (PC) receive androgen deprivation therapy (ADT) [1] and commonly experience adverse side effects (fatigue, decreased musculoskeletal and cardiorespiratory fitness, low mood, body composition changes, and reduced quality of life (QOL)) [2–4]. Exercise is one of the most effective interventions to counter ADT side effects and systematic reviews confirm exercise is safe and associated with psychophysiological benefits including improved QOL, strength, and aerobic capacity, and less fatigue) [5–8].

While benefits have been frequently observed with 1:1 supervised programs [7], few studies have directly compared exercise delivery methods. A review that suggested greater improvements with supervised group-based versus home-based training [7] was limited to indirect comparisons [7] and one randomized controlled trial (RCT) that directly compared supervised personal versus group training was underpowered (*n* = 13) and without a home-based arm [9]. And while exercise delivers significant benefits to men on ADT, it is unclear whether there is a difference in cost effectiveness and outcomes between different delivery approaches of exercise.

The present pilot non-inferiority trial compared 1:1, site-based personal training (PT) with two less-resource-intense approaches: group, site-based training (GROUP) and individual home-based training (HOME). Our primary aims were to: (i) determine the feasibility of conducting a large multi-center non-inferiority RCT of three exercise delivery models in men with PC on ADT; (ii) obtain preliminary efficacy estimates for (a) GROUP, and (b) HOME exercise programs, compared to PT on the clinical outcomes of QOL and physical fitness, and (iii) select a primary outcome and intervention arms for a phase III trial.

## Methods

### Study design

This randomized phase II non-inferiority trial recruited patients from two Canadian academic tertiary care centers – the Princess Margaret Cancer Centre (Toronto) and the Tom Baker Cancer Centre (Calgary). Ethics approval was obtained at both institutions and all participants provided informed consent. The trial was registered at clinicaltrials.gov (Registration #NCT02046837). The detailed trial protocol was published [10] and is summarized below.

### Study participants

Eligible participants were diagnosed with PC of any stage; starting or continuing on ADT for at least 6 months (or who remained biochemically castrate after stopping ADT); able to communicate in English; and living in proximity to a study centre. Each potential participant was screened with the Physical Activity Readiness questionnaire (PAR-Q+ or PARmed-X) [11] and/or received physician clearance to participate. We excluded men already engaging in 150 min of weekly moderate to vigorous physical activity (MVPA) or who had a condition interfering with their ability to participate [10].

### Randomization

Following the baseline assessment, participants were randomized equally (1:1:1) to PT, GROUP, and HOME. Randomization used varying block sizes and was stratified by duration of prior ADT use (< 3 months versus ≥3 months) [12] and site. The randomization sequence was created by a biostatistician using computer-generated random numbers and participants were allocated to treatments via a custom-built website, ensuring allocation concealment.

### Intervention

The 6-month exercise intervention consisted of three exercise delivery arms: PT, GROUP, and HOME, which have been described in detail [10]. All programs were prescribed in accord with the FITT principle: Frequency, Intensity, Time, and Type and were individualized per baseline fitness assessment results. This is detailed in our protocol paper for the phase II trial [10]. Participants were asked to complete 4–5 days per week of mixed modality exercise incorporating aerobic, resistance, and flexibility training. The target time and relative workload (target heart rate 60–70% of heart rate reserve) were consistent across interventions. Exercise intensity was monitored using the 10-point Rating of Perceived Exertion (RPE) scale. Participants maintained their intensity level between an RPE of 3 and 6 during exercise sessions. HR monitors (Polar, NY, USA) were used at 3-week intervals in each intervention arm to ensure that participants reached their target heart rate range, calibrating with the RPE scale. HOME participants were trained to use HR monitors, which were provided to them for the intervention period. If a participant’s HR is outside of his target HR range, exercise intensity will be modified to ensure training within the target HR zone. Participants who need to increase their aerobic exercise workload will first increase exercise duration (e.g. walking minutes), followed by the intensity of exercise (e.g., walking speed). If a participant is able to perform ≥12 repetitions and 3 sets of any given resistance training exercise, the resistance level used for that exercise will be increased (e.g., from a medium to a heavy band). All participants received a study manual outlining exercise techniques and safety principles as well as further details on specific resistance and flexibility exercises they were prescribed with as part of their intervention. These resistance exercises include variations of stability ball wall squat, hamstring curl, push-ups, seated row, biceps curl, triceps extension, upright row, and plank. Participant progression was individualized and monitored by a Certified Exercise Physiologist (CEP) or health coach every 3 weeks. Exercise adherence was documented on standardized forms used at both study sites.

Each program also included an education component that consisted of 12 topics focusing on common issues facing new exercisers (Additional file 1: Table S1). These topics were informally discussed with 1:1 participants during their supervised sessions for PT, and during weekly phone calls for HOME. Similarly these education topics were also discussed with GROUP during and in between exercises. Discussions typically lasted 10–20 min per topic.

### Personal training

Participants in the PT group completed 3 sessions/week in a dedicated gym space and were encouraged to perform 1–2 additional days/week of independent (home-based) exercise. Each session was supervised by a CEP and consisted of aerobic training (15–30 min), resistance training (with a focus on major muscle groups), and flexibility training (5–10 min of static stretching at the end of each session). All participants were provided with resistance bands to support independent exercise.

### Group-supervised training

This protocol mirrored that of the PT protocol described above, and was delivered in a small group format (4–6 individuals per group), supervised by a CEP, in the same gym area but at separate times from the PT group.

### Home-based training

The identical protocol to PT and GROUP was implemented in HOME. Participants received resistance bands, stability ball, exercise mat, HR monitor, and smartphone with a 6-month paid plan to connect with a health coach and access specific health software (Connected Wellness Platform, NexJ Systems, Inc.). Weekly health coach (by smartphone) communications via text and voice reviewed exercise sessions, guidance, and helped with smartphone applications. Additional details are described in the protocol [10].

### Outcome assessments

Outcome assessments were completed at baseline, 3, and 6 (end of intervention) months. Additional assessments at 9 and 12 months were completed post-intervention, and will be reported separately. Blinded outcome assessments were conducted by a CEP.

### Feasibility outcomes

We assessed recruitment rate, retention rate, adherence, outcome capture, and satisfaction/barriers. Definitions and measurement details are as follows:

#### Recruitment rate

We recorded the number of patients approached and the percentage of these patients recruited. We documented reasons for non-participation.

#### Retention rate

Retention was assessed by measuring attrition throughout the intervention period and at each assessment time point. Reasons for drop-out were recorded.

#### Adherence

Given the trial’s feasibility focus, three elements of adherence (self-reported MPVA, accelerometry-based MPVA, sessional attendance) are reported. Self-reported MVPA was obtained from the Godin Leisure-Time Activity Questionnaire (GLTEQ) [13, 14], a measure of weekly physical activity (PA) in which respondents report the amount of time (in 15-min increments) that is spent on light, moderate, and strenuous physical activity each week. Objective physical activity was measured using accelerometers (Actigraph GT3X, Pensacola, FL) worn for 7 days while awake at each outcome assessment time point [15, 16]. Data were extracted from the accelerometer in 60-s epochs and were screened to ensure i) at least 4 days of valid data, (ii) at least 10 h of wear time per day; (iii) non-wear time will be assessed as periods of time with no movement (0 counts per minute) for more than 1 h at a time. MVPA is defined as activity > 1952 counts per minute [17]. Finally, attendance at supervised exercise sessions (for 1:1 and group-supervised intervention arms) was collected.

For MVPA outcomes, our target was at least 150 min of MVPA per week [18, 19] for at least 70% of the intervention period.

#### Outcome capture

Given the importance of minimizing missing data [20], we examined whether outcome measures could be successfully collected at each time point.

#### Satisfaction/barriers

Satisfaction surveys were administered to participants that assessed barriers/challenges to participation, and feedback on how to improve delivery. A questionnaire modified from a previous trial was used [21] and it included a 10-point Likert scale rating of the exercise program and a 5-point Likert scale rating of overall study satisfaction.

### Clinical efficacy outcomes (listed in Additional file 2: Table S2)

#### QOL and fatigue

Health-related QOL was assessed using the Functional Assessment of Cancer Therapy – General (FACT-G) [22, 23]. The Functional Assessment of Cancer Therapy – Prostate (FACT-P) evaluated prostate-specific QOL [24] and the Functional Assessment of Cancer Therapy – Fatigue (FACT-F) was used to evaluate cancer-related fatigue [25]. All three measures have established psychometric characteristics and have been used in multiple exercise trials in prostate cancer [5–8].

#### Physical fitness

Aerobic fitness was directly assessed with the modified Bruce treadmill protocol (graded exercise test) [18] which measures volitional peak oxygen consumption (VO_2_ peak) using a metabolic cart. Upper body strength was assessed by grip strength, measured with a Jamar dynamometer [26]. Lower body functional capacity was measured with the 1-min sit-to-stand test [27, 28]. These measures are relevant to this population as men on ADT have significantly reduced muscle strength for upper and lower body as well as impaired functional performance compared to controls [4, 12, 29]. We chose a lower body functional capacity measure rather than traditional measures of leg strength as the former is more clinically relevant to older patients and more feasible to measure in terms of physical space, equipment costs, and participant burden.

### Safety

Safety procedures are described elsewhere [10]. Adverse events were documented using the National Cancer Institute common terminology criteria for adverse events v4.0 [30].

### Cost-effectiveness analysis

This study investigated the feasibility of completing a companion economic evaluation (as part of a subsequent phase III trial). Although there was no formal cost-utility analysis in this trial, relevant outcomes and health status (utilities) were collected at each time point using the European QOL 5-Dimension 3-Level measure (EQ-5D-3 L) [31]. Costs were also captured with a patient-reported diary [32].

### Sample size calculation/power

Following standard guidelines for a phase II RCT [33, 34], we determined that a sample size of 30 patients per arm (90 patients in total) would provide sufficiently precise estimates of parameters related to important feasibility information as well as the primary clinical outcomes that would be crucial to planning a phase III study. Assuming a drop-out rate of 10%, our goal was to recruit 100 patients.

### Statistical analysis

Statistical analysis focused on feasibility outcomes, namely estimation of recruitment and retention rates and adherence. In addition, reasons for not participating in the trial were documented and tallied. Descriptive statistics were used for each of these feasibility measures.

QOL, fatigue, and fitness outcomes were analyzed using Bayesian linear mixed effects model with subject-specific random intercepts, fixed effects for time and group, and group-by-time interactions. Models were fitted through rjags in R version 3.4.0. Both the GROUP and HOME arms were compared to PT. Mean differences in baseline to 6-month change and their 95% credible intervals (CrIs) were calculated, along with the posterior probability of inferiority that the mean difference lay outside the pre-specified non-inferiority margin for the specific outcome. Diffuse normal priors were used for all regression coefficients, and uniform prior distributions with a large upper bound were used for the standard deviations of the residual error and between-subject random effects. After a burn-in of 5000 iterations, 10,000 further samples were collected from each of three parallel chains and used for inference. Convergence was assessed with the Gelman diagnostic.

### Criteria to move forward to phase III RCT

A priori, we defined the following criteria to move forward to a phase III trial: recruitment rate of at least 25%, adherence and retention rates of 70%, moderate or greater participant satisfaction, and at least 80% data capture of clinical outcomes.

## Results

### Baseline characteristics

Sixty-five men were enrolled (45 in Toronto, 20 in Calgary) between December 19, 2013 and October 31, 2015. Follow-up occurred between June 2013 and October 2016. Six men withdrew before randomization. Baseline characteristics for 59 subjects (mean age 70) are presented in Table 1; groups were similar for sociodemographic, clinical, and outcome measures.

**Table 1:** Baseline characteristics of study participants

Variable		1:1 (*n* = 19)	Group-Supervised (*n* = 16)	Home-Based (*N* = 18)	*P*-value
Age (years), mean (SD)		69.2 (7.3)	71.5 (7.2)	69.6 (8.1)	0.59
Education, post-secondary, n (%)		14 (73.7)	15 (93.8)	11 (61.1)	0.085
Race, White, n (%)		12 (63.2)	14 (87.5)	12 (66.7)	0.59
Marital Status, married (%)		13 (68.4)	14 (87.5)	10 (55.6)	0.63
Working Status, retired (%)		11 (57.9)	9 (56.3)	13 (72.2)	0.44
Smoking Status, never smoked (%)		6 (31.6)	8 (50.0)	7 (38.9)	0.52
ECOG Performance Status, 0–1(%) Missing		17 (100.0)	15 (100.0)	14 (92.7)	0.64
		2	1	3	
Karnofsky Score, mean % (SD)		81 (11.1)	87 (9.9)	79 (9.2)	0.082
PSA at diagnosis, ng/mL, median (IQR)		9 (7–20)	10 (8–17)	15 (6–35)	0.34
Charlson Comorbidity score, n (%)	0	11 (57.9)	13 (81.3)	15 (83.3)	0.23
	1	6 (31.6)	3 (18.7)	0	
	≥2	1 (5.3)	0	1 (5.5)	
	Missing	1 (5.3)	0	2 (11.0)	
Clinical Stage, n (%)	T1-T2	9 (56.3)	9 (60.0)	9 (52.9)	0.99
	T3+	6 (37.5)	5 (33.3)	7 (41.2)	
	Missing	4	2	2	
Gleason score, n (%)	6	3 (21.4)	1 (6.7)	1 (5.9)	0.18
	7	4 (28.6)	4 (26.7)	10 (58.8)	
	8–10	7 (50.0)	10 (66.7)	6 (35.3)	
	Missing	5	1	1	
Duration of ADT, n (%)	< 3 mo.	5 (26.3)	2 (16.7)	6 (33.3)	0.59
	≥3 mo.	14 (73.7)	10 (83.3)	12 (66.7)	
	Missing	0	4	0	
Indication for ADT, n (%)	Adjuvant	10 (52.6)	7 (43.8)	8 (44.4)	0.62
	Biochemical relapse	3 (15.8)	5 (31.3)	7 (38.9)	
	Metastases	2 (10.5)	2 (12.5)	0	
	Unknown	4 (21.1)	2 (12.5)	3 (16.7)	
FACT-G (total), mean (SD)		84.2 (17.5)	85.9 (10.4)	85.4 (12.3)	0.93
FACT-P (total), mean (SD)		118.6 (23.9)	119.9 (14.5)	120.4 (17.8)	0.96
FACT-Fatigue, mean (SD)		41.0 (10.4)	39.5 (8.1)	39.1 (11.0)	0.84
VO2peak (L/min), mean (SD)		1.7 (0.4)	1.9 (0.5)	1.8 (0.5)	0.81
Grip strength, mean (SD)		28.6 (5.7)	32.6 (8.3)	33.9 (10.3)	0.13
Timed chair stands, mean (SD)		24.5 (7.7)	24.8 (10.0)	23.7 (6.3)	0.90

### Feasibility outcomes (Table 2)

#### Recruitment and retention

**Fig. 1:**
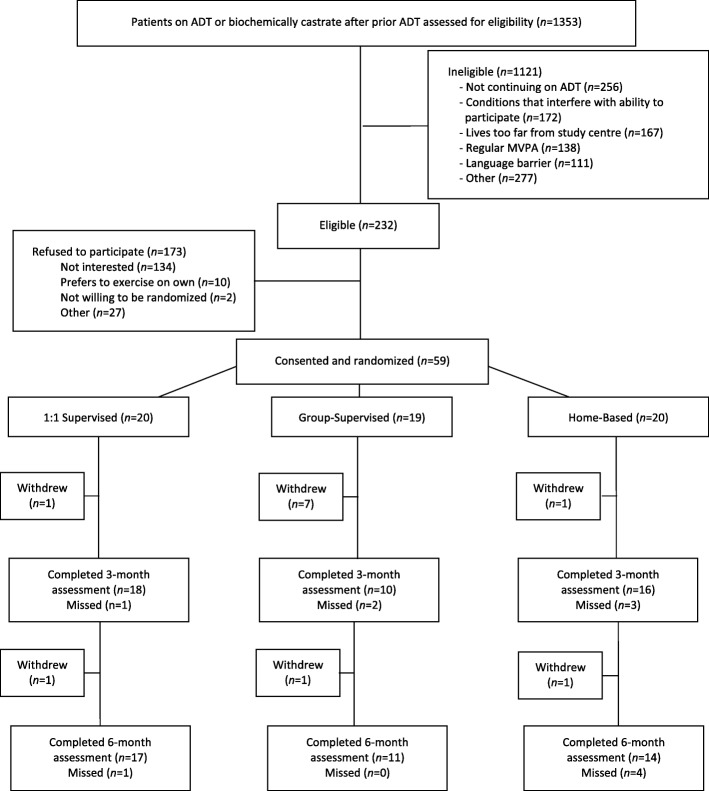
This shows the flow of patients throughout the study following CONSORT guidelines

Participant flow is detailed in Fig. 1. Among all participants assessed for eligibility (*n* = 1353), 1121 were ineligible. The most common reasons for ineligibility were not continuing on ADT (*n* = 256), having one or more conditions that precluded trial participation (*n* = 172), living too far from a study centre (*n* = 167), and currently meeting MVPA guidelines (*n* = 138). Additional reasons are shown in Fig. 1. Among the 232 eligible participants, 173 declined participation, and 59 consented (recruitment rate 25.4% of eligible participants). The most common reason for declining participation was lack of interest (*n* = 134). Other reasons for declining participation are shown in Fig. 1. Recruitment was slower than anticipated and the trial closed prematurely.

**Table 2:** Summary of feasibility outcomes

Outcome	All Participants *n* = 59		
Recruitment	25.4%		
Retention	76.3%		
Outcome capture			
Quality of life measures, 6 months	80%		
Physical performance measures^a^, 6 months	91%		
VO_2_ peak, 6 months	57%		
Satisfaction (4 or higher on 5-point Likert scale)	88%		
Outcome	PT *n* = 20	Group *n* = 19	Home *n* = 20
Adherence			
Supervised sessions attended	75%	71%	N/A
MVPA by Godin, 6 months	53%	30%	31%
MVPA by accelerometry, 6 months	42%	22%	50%
Safety			
Grade 1 events, n	1	0	0
Grade 2 events, n	0	0	2
Grade 3+ events, n	0	0	0

*MVPA* moderate to vigorous physical activity, *PT* personal training

^a^Includes both grip strength and 60-s chair stands

Retention was 76.3% for the 6-month study duration and did not differ between groups (data not shown).

#### Adherence

Attendance at supervised exercise sessions was 75% and 71%, respectively (PT and GROUP arms). Self-reported MVPA of at least 150 min/week was reported by 53% of participants in PT, 31% in HOME and 30% in GROUP at the 6–month time point. Using accelerometry, 50% of participants in HOME, 42% in PT, and 22% in GROUP met MVPA guidelines at 6 months (see Table 2 and Additional file 3: Table S3). Random audits of supervised exercise session logs showed that over 90% of men achieved target intensity and duration during sessions (data not shown). The most common reasons for missing a supervised exercise session were due to travel, personal/family illness, or work commitments (data not shown).

#### Outcome capture and satisfaction

Completion of functional fitness measures (grip strength, chair stands) at baseline, 3, and 6 months was high (100, 87, and 91%, respectively). However, VO_2_ peak test completion was much lower, averaging 61% across the 3 assessment time points, due to both participant preference (several refused to perform the test) and clinical reasons (primarily lower extremity arthritis). QOL and fatigue measures were completed by 84, 72, and 80% of participants at baseline, 3, and 6 months, respectively. Eighty-eight percent of participants rated overall trial satisfaction at 4 or above (‘Very Satisfied’) on a 5-point Likert scale. There were no differences in outcome capture or satisfaction per intervention arm (data not shown).

#### Cost and utility data completion rates

Return rates for the EQ-5D ranged from 82 to 88% over the three time points and did not vary by group (data not shown). Cost diaries were returned by 84–91% over the three time points, with complete data in 74–80% of returned diaries (Additional file 4: Table S4).

### Clinical efficacy outcomes

#### QOL and fatigue

**Fig. 2:**
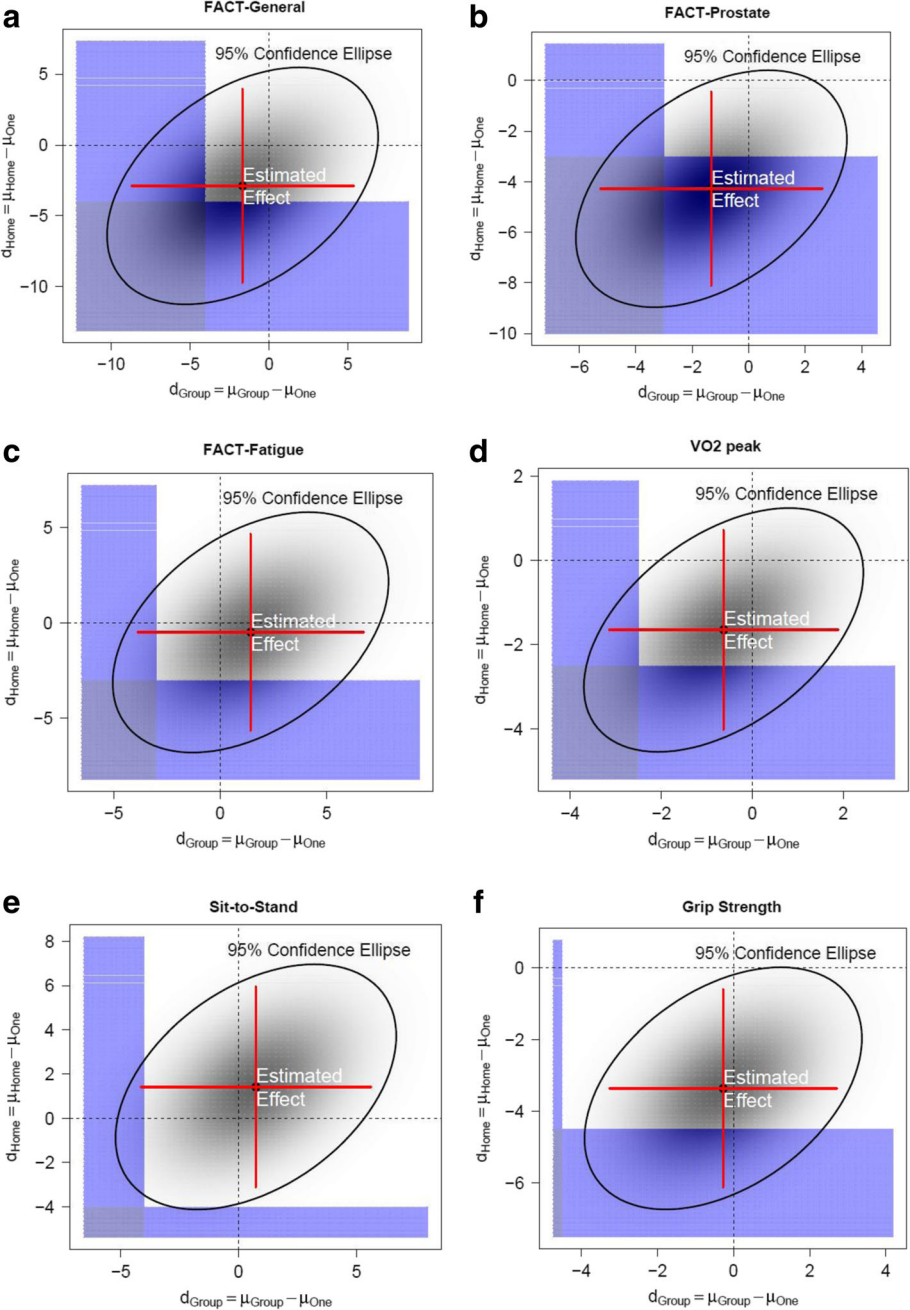
This shows the probability of inferiority of group-based (horizontal axis) and home-based (vertical axis) arms compared to the personal training arm for each of six outcomes (FACT-General (Panel **a**), FACT-Prostate (Panel **b**), FACT-Fatigue (Panel **c**), VO2 peak (Panel **d**), Sit-to-Stand (Panel **e**), Grip Strength (Panel **f**)) . The ellipse shows the 95% credible interval around the estimated effect. The light purple shaded areas represent inferiority regions that are larger than the minimum specified inferiority boundary for the specific outcome for one arm (either group-based or home-based), whereas the dark purple shaded areas represent inferiority regions where both arms are inferior to the personal training arm. See text and supplemental methods for more details

The change from baseline to 6 months in the FACT-P was 4.3 points worse in HOME than in PT (95% CrI -8.1 to − 0.5, probability of inferiority = 74%). In comparison, for GROUP it was − 1.4 (95% CrI -5.4 to 2.6, probability of inferiority = 21%). For FACT-G the change from baseline to 6 months was 2.9 points worse for HOME and 1.7 points worse for GROUP than PT, with the probability of inferiority being 38 and 26%, respectively. Changes in FACT-F were similar between arms (Table 3) and Fig. 2.

**Table 3:** Between group efficacy outcomes

Outcomes	Group-supervised versus PT	Home-based versus PT	Non-inferiority margin	Probability of inferiority of Group-supervised to PT^a^	Probability of inferiority of Home-based to PT^a^
Quality of Life and Fatigue					
FACT-G	−1.7 (−8.7 to 5.4)	−2.9 (−9.7 to 3.8)	4 points	25.6%	37.9%
FACT-F	1.5 (−3.9 to 6.6)	−0.5 (−5.9 to 4.8)	3 points	4.9%	17.6%
FACT-P	−1.4 (−5.4 to 2.6)	−4.3 (− 8.1 to −0.5)	3 points	20.9%	74.4%
Physical Fitness					
VO_2_ peak	− 0.7 (− 3.2 to 1.8)	− 1.8 (−4.2 to 0.6)	2.5 mL/kg/min	8.2%	26.7%
Grip strength	−0.3 (− 3.3 to 2.7)	− 3.4 (−6.3 to − 0.6)	4.5 kg	0.2%	23.3%
Sit-to-stand	0.7 (− 4.3 to 5.5)	1.4 (− 3.3 to 5.9)	4 repetitions	3.1%	1.1%

Note: The above values are mean differences between baseline and 6 months with 95% confidence intervals in parentheses. The reference group is the personal training arm

*FACT* Functional Assessment of Cancer Therapy (*G* general, *F* fatigue subscale, *P* prostate); *PT* personal training

^a^The Bayesian posterior probability of inferiority is calculated as the probability that the mean outcome in the comparator arm is lower than that in the personal training arm by at least the specified non-inferiority margin. See text for more details

#### Physical fitness

Participants in HOME had grip strength changes between 0 and 6 months that were 3.4 kg worse than those in PT (95% CrI -6.3 to − 0.6, probability of inferiority = 23%). Similarly, changes in VO_2_ peak were − 1.8 mL/kg/min worse in HOME compared to PT, probability of inferiority = 27%. For other fitness measures (chair stands for HOME and all three physical fitness measures for GROUP), changes were similar between groups (Table 3) and Fig. 2.

#### Non-inferiority

Overall, two of six outcomes had a > 20% probability of being inferior for GROUP compared to PT. In contrast, four of six outcomes for HOME had a > 20% probability of being inferior to PT (Table 3).

#### Safety

No grade 3 or higher adverse effects occurred. Three adverse events were reported (two grade 2 events in HOME participants and one grade 1 event in a PT participant; primarily musculoskeletal) (Table 2) and Fig. 2.

## Discussion

Increasing evidence suggests exercise-related QOL and fitness benefits for men with PC on ADT. Although robust data support PT [5–8], it is resource intensive and unlikely to be as cost-effective as home or group-based programming. As public funding is unlikely with such expense, we must test lower resource alternatives. Yet exercise intervention is needed, as most PC men on ADT are physically inactive and risk numerous side effects [35, 36].

In the present trial, the feasibility and efficacy of three training models (personal training, supervised group training, and home-based training) were investigated. From a feasibility perspective, recruitment rates were somewhat lower than expected (~ 25%), and at the lower end of recruitment rates for published exercise trials in men with prostate cancer [37–43]. Retention was acceptable in all three arms. Although adherence to supervised exercise sessions was high, independent exercise adherence was considerably lower. This has at least three implications for future studies. First, greater attention needs to be focused on regular monitoring of adherence throughout the trial, particularly for home-based strategies. Second, predictors of adherence need to be understood to help guide the selection of strategies to improve adherence. Two studies in this population have suggested that age, role functioning and hormonal symptoms, intention, and exercise stage of change were predictors of adherence [44, 45]. How to target these factors is much less clear. Third, an important challenge in supervised programs is that they are all time-limited, and understanding how people who were enrolled in supervised exercise interventions transition to independent exercise is key to ensuring long-term exercise adherence. Additional work examining ways of increasing recruitment by employing more efficient ways of identifying eligible participants, making the study available in more locations and with varied supervised exercise session timings, modifying eligibility criteria, or by exploring less intensive physical activity programs may be valuable.

Clinical efficacy measures were collectable in most patients, but directly measured peak VO_2_ was difficult for many subjects, prompting the consideration of alternative tests in future trials that are less burdensome and more clinically relevant for a mostly sedentary older population. Finally, satisfaction with the study was high in all groups.

Although most efficacy outcomes were similar between GROUP and PT participants, slow recruitment led to lower power than planned and large credible intervals for specific estimates. This reduced the ability to determine whether HOME or GROUP was significantly worse than PT. Importantly, while four of six outcomes had a probability > 20% of being inferior in HOME (vs. PT), only two outcomes had a substantially greater probability of inferiority. However, it is interesting to note a numerical suggestion from accelerometry data that the highest MVPA was achieved in HOME. These discrepancies may be due to the walking emphasis (in the home-based arm), necessitating closer physical activity monitoring (via wearable technologies) and more emphasis on diverse exercises in the future. Given the theoretical advantages of substantially reduced costs, ease of scalability, and greater long-term adherence with home-based programs, our findings can best be viewed as supporting the need for a larger study incorporating refinements to improve recruitment and adherence while collecting relevant clinical and costing data. Post-intervention phase adherence data will also be vital to capture.

Important study strengths were a randomized design with concealed allocation and blinded outcome assessment to directly compare, for the first time, the three prominent exercise delivery models with outcomes that answer important questions. Limitations included slow recruitment and the challenge of adherence with intensive behavioural/lifestyle interventions that requires further innovation to foster sustained change. Generalizability is also a challenge since fewer than 30% of eligible men participated.

## Conclusions

In summary, our results suggest that less resource-intensive exercise programs may provide benefits to QOL and fitness similar to those of the gold-standard 1:1 supervised exercise program and require further study in larger more diverse samples, although recruitment and adherence issues need to be addressed. While our findings suggested that benefits in clinical outcomes may be attenuated with home-based programs compared to group-based programs, our trial was not designed or powered to address this directly and the key dimension of cost-effectiveness (which may be most favorable for the home-based program) requires consideration. Based on the feasibility and efficacy data of this trial, a larger trial and companion cost-effectiveness analysis can further advance understandings of the value of alternative exercise programming in accord with at least three suggested trial modifications: (a) replacement of the VO_2_ peak with the 6-min walk test [46], a submaximal aerobic test more functionally relevant to older populations; (b) addition of wearable technology to provide daily MVPA monitoring [47, 48]; (c) inclusion of additional centres and strategies to improve recruitment and generalizability.

## Additional files

Additional file 1: **Table S1.** Table listing education topics covered in all three intervention arms. (DOCX 15 kb)

Additional file 2: **Table S2.** Summary of study measures at specified time points. (DOCX 15 kb)

Additional file 3: **Table S3.** Table describing study participant adherence in all three intervention arms. (DOCX 15 kb)

Additional file 4: **Table S4.** EQ-5D and Health Care Costs Diary Feasibility Data. (DOCX 14 kb)

## Abbreviations

ADT: Androgen deprivation therapy
CEP: Certified exercise physiologist
CrI: Credible interval
EQ-5D-3 L: European Quality of Life 5 dimension 3 level
FACT-F: Functional Assessment of Cancer Therapy – Fatigue subscale
FACT-G: Functional Assessment of Cancer Therapy – General
FACT-P: Functional Assessment of Cancer Therapy – Prostate-specific subscale
MVPA: Moderate to vigorous physical activity
PA: Physical activity
PAR-Q: Physical activity readiness questionnaire
PC: Prostate cancer
PT: Personal training
QOL: Quality of life
RCT: Randomized controlled trial
RPE: Rating of perceived exertion
