# Supplementary material for: A phase II randomized controlled trial of three exercise delivery methods in men with prostate cancer on androgen deprivation therapy
Source: BMC Cancer. 2019 Jan 3;19:2. doi: 10.1186/s12885-018-5189-5 (PMC6318980; doi:10.1186/s12885-018-5189-5)
Supplement: Supplementary file 1 — Table S1. Table listing education topics covered in all three intervention arms. (DOCX 15 kb) [file 12885_2018_5189_MOESM1_ESM.docx]

**Table S1: Educational Topics**

| Week 1 | Introduction to Exercise |
| --- | --- |
| Week 2 | Goal Setting |
| Week 3 | Behaviour Change |
| Week 4 | Planning for Barriers |
| Week 5 | Social Support |
| Week 6 | Monitoring Behaviour |
| Week 7 | Maintaining Motivation |
| Week 8 | Personal Control |
| Week 9 | Self-Reward, Discipline & Attitude |
| Week 10 | Adapting Your Program |
| Week 11 | Health and the Media |
| Week 12 | Lifelong Active Living |

These topics were covered in all three intervention arms.
