# Supplementary material for: A phase II randomized controlled trial of three exercise delivery methods in men with prostate cancer on androgen deprivation therapy
Source: BMC Cancer. 2019 Jan 3;19:2. doi: 10.1186/s12885-018-5189-5 (PMC6318980; doi:10.1186/s12885-018-5189-5)
Supplement: Supplementary file 2 — Table S2. Summary of study measures at specified time points. (DOCX 15 kb) [file 12885_2018_5189_MOESM2_ESM.docx]

**Table S2: Summary of Study Measures at Specified Time Points**

| **Domain/Measure** | **Time required** | **T_0_: (Baseline)** | **T_1_:**  **(3 mo.)** | **T_2_:6 mo.**  **(End Int.)** |
| --- | --- | --- | --- | --- |
| **Quality of life**  FACT-G  FACT-P  FACT-F | 8-10 min  4-5 min  5 min | ●  ●  ● | ●  ●  ● | ●  ●  ● |
| **Physical Fitness**  VO_2_ Peak  Sit-to-Stand Test  Grip Strength | 20 min  1 min  1 min | ●  ●  ● | ●  ●  ● | ●  ●  ● |
| **Adherence** |  |  |  |  |
| Accelerometer | **-** | ● | ● | ● |
| GLTEQ (weekly)  Sessional attendance* | <5 min  - | ● | ● | ● |
| **Cost-Effectiveness** |  |  |  |  |
| Health questionnaire | 5-10 min | ● | ● | ● |
| EQ-5D | 5 min | ● | ● | ● |

* Only for those in supervised groups (done weekly)

**Abbreviations:** EQ-5D = EuroQol 5 dimensions of health scale; FACT-G = Functional Assessment of Cancer Therapy General; FACT-F = Fatigue subscale; FACT-P = Prostate subscale; GLTEQ = Godin Leisure Time Exercise Questionnaire; VO2 Peak = Peak Volume of Oxygen Consumption
