# Supplementary material for: A phase II randomized controlled trial of three exercise delivery methods in men with prostate cancer on androgen deprivation therapy
Source: BMC Cancer. 2019 Jan 3;19:2. doi: 10.1186/s12885-018-5189-5 (PMC6318980; doi:10.1186/s12885-018-5189-5)
Supplement: Supplementary file 3 — Table S3. Table describing study participant adherence in all three intervention arms. (DOCX 15 kb) [file 12885_2018_5189_MOESM3_ESM.docx]

**Table S3: Adherence Measures**

|  | **Baseline** | | | **0-3 Months** | | | **0-6 Months** | | |
| --- | --- | --- | --- | --- | --- | --- | --- | --- | --- |
|  | ***1:1*** | ***Group*** | ***Home*** | ***1:1*** | ***Group*** | ***Home*** | ***1:1*** | ***Group*** | ***Home*** |
| **Accelerometry,**  **% with ≥150 min MVPA** | 42.1 | 9.1 | 43.8 | 31.3 | 44.4 | 18.2 | 41.7 | 22.2 | 50.0 |
| **Godin LTEQ,**  **% with ≥150 min MVPA** | 11.1 | 0.0 | 13.3 | 68.8 | 40.0 | 50.0 | 53.3 | 30.0 | 30.8 |
| **Attendance at supervised exercise sessions,**  **mean %** | N/A | N/A | N/A | 83.6* | 78.3* | N/A | 74.4 | 63.2 | N/A |

*Currently represents Toronto data only

LTEQ = Leisure Time Exercise Questionnaire; MVPA = moderate to vigorous physical activity

Note: Participants in the 1:1 arm of the intervention did most of their aerobic exercise on a recumbent bike. This exercise, along with their resistance work would likely not be accurately captured via accelerometry versus those in the group arm of the intervention who participated in circuit-based classes that would be better captured via accelerometry. Participants in the home arm completed various exercises to meet their goals since their exercise was done in an independent setting. Accelerometry and adherence data collected at the 6 month time point was done so following the completion of the active intervention (i.e. after supervised exercise sessions were completed).
