# Supplementary material for: A phase II randomized controlled trial of three exercise delivery methods in men with prostate cancer on androgen deprivation therapy
Source: BMC Cancer. 2019 Jan 3;19:2. doi: 10.1186/s12885-018-5189-5 (PMC6318980; doi:10.1186/s12885-018-5189-5)
Supplement: Supplementary file 4 — Table S4. EQ-5D and Health Care Costs Diary Feasibility Data. (DOCX 14 kb) [file 12885_2018_5189_MOESM4_ESM.docx]

| **Time point** | **Any part of Assessment Complete** | **EQ-5D Total** | **Complete** | **Incomplete** |
| --- | --- | --- | --- | --- |
| Baseline | 58 | 48 (83%) | 46 (79%) | 2 (VAS) |
| 3 Months | 44 | 36 (82%) | 34 (77%) | 2  (1 with 5 items missing; 1 left blank but other questionnaires complete) |
| 6 Months | 43 | 38 (88%) | 38 (88%) | 0 |

**Table S4: EQ-5D and Health Care Costs Diary Feasibility Data**

**Data Summary**

**Health Care Costs Diary Data Summary**

| **Time point** | **Any part of Assessment Complete** | **Costs Diary Returned** | **Costs Diary Complete** |
| --- | --- | --- | --- |
| Baseline | 58 | 49 (84%) | 39 (80%) |
| 3 Months | 44 | 37 (84%) | 29 (78%) |
| 6 Months | 43 | 39 (91%) | 29 (74%) |
